# Telemedicine use by pediatric rheumatologists during the COVID-19 pandemic

**DOI:** 10.1186/s12969-021-00565-7

**Published:** 2021-06-16

**Authors:** Rajdeep Pooni, Tova Ronis, Tzielan Lee, the CARRA Investigators

**Affiliations:** 1 Stanford Cildren’s Health, Stanford University School of Medicine, Palo Alto, California, USA; 2 Children’s National Hospital, Washington D.C; George Washington School of Medicine & Health Sciences, Washington DC, USA

**Keywords:** Telemedicine, Telehealth, Healthcare delivery services, COVID-19

## Abstract

**Background:**

To characterize various aspects of telemedicine use by pediatric rheumatology providers during the recent pandemic including provider acceptability of telehealth practices, clinical reliability, and clinical appropriateness.

**Methods:**

An electronic survey was generated and disseminated amongst the Childhood Arthritis and Rheumatology Research Alliance (CARRA) listserv (*n* = 547). Survey items were analyzed via descriptive statistics by question.

**Results:**

The survey response rate was 40.8% (*n* = 223) with the majority of respondents in an attending-level role. We observed that musculoskeletal components of the exam were rated as the most reliable components of a telemedicine exam and 86.5% of survey respondents reported engaging the patient or patient caregiver to help conduct the virtual exam. However, 65.7% of providers reported not being able to elicit the information needed from a telemedicine visit to make a complete clinical assessment. We also noted areas of disagreement regarding areas of patient engagement and confidentiality. We found that approximately one-third (35.8%) of those surveyed felt that their level of burnout was increased due to telemedicine.

**Conclusion:**

In general, providers found exam reliability (specifically around focused musculoskeletal elements) in telemedicine visits but overall felt that they were unable to generate the information needed to generate a complete clinical assessment. Additionally, there were suggestions that patient engagement and confidentiality varied during telemedicine visits when compared to in-person clinical visits. Further qualitative work is needed to fully explore telemedicine use in pediatric rheumatology.

**Supplementary Information:**

The online version contains supplementary material available at 10.1186/s12969-021-00565-7.

## Background

The coronavirus disease 19 (COVID-19) pandemic has radically impacted the way health systems operate across the United States and around the world. One of the most visible impacts has been in ambulatory care centers with the rapid deployment of telemedicine use to deliver both routine and urgent care, with centers reporting hundreds-fold increases in daily telemedicine visits [1]. Historically, telemedicine has been a modality of care to help improve access and establish continuity of care, but the uptake prior to the pandemic was modest at best [2]. Due to the recent pandemic and accompanying telehealth policy changes [3], the volume and attitudes around telemedicine have been shifting. Pediatric rheumatologists are faced with multiple issues in health care delivery and availability [4] and a few reports have begun to explore telemedicine within this specialty in regards to patient acceptability [5] along with cost and time effectiveness [6]. The historical paucity of telemedicine adoption within this specialty may be for a number of reasons including the reliance on patient-reported outcomes in addition to objective clinical findings [7] and lack of standardization of telemedicine exam practices [8].

With the rapid implementation of telemedicine over the previous several months, there is a unique opportunity to evaluate telemedicine use in the pediatric rheumatology setting. Recent pediatric studies have described the ability to deploy telemedicine rapidly during the COVID-19 pandemic [9], however, few studies have described the potential benefits and clinical limitations from the subspecialists’ perspective during this time. Though clinicians and patients are clearly impacted by COVID-19 in multiple ways, this study explores the perceived limitations and benefits of the teleclinical interaction and provider acceptability of various telehealth practices. This includes rheumatologists’ perceptions regarding specific areas of clinical assessment such as the exam and appropriateness of telemedicine follow-up by visit type, as well as potential patient-facing issues including engagement and confidentiality. It is critical that we understand this baseline state of telemedicine use amongst pediatric rheumatologists so that we can more safely and effectively integrate its use in routine care.

## Methods

The Childhood Arthritis and Rheumatology Research Alliance (CARRA) is a collaborative research network inclusive of pediatric rheumatologists, pediatric rheumatology trainees, advanced practice professionals, nurses, researchers, and patient partners with members primarily in the United States and Canada. A survey was developed with a goal of evaluating various aspects of telehealth use (please see Supplementary Data). The study was approved by the Stanford Institutional Review Board. The electronic survey (SurveyMonkey Inc., San Mateo, CA) was disseminated via the CARRA provider listserv during June and July 2020. Survey results were analyzed via descriptive statistics and other trends in data were evaluated using one-way ANOVA tests in SAS University Edition (SAS Institute, Cary, NC).

## Results

### Responses and participants

A total of *n* = 547 surveys were sent via the CARRA member listserv and 40.8% (*n* = 223) of subjects agreed to participate in the study. The majority of participants were pediatric rheumatologists (71.6%) followed by fellow trainees (16.4%) but included other professionals such as nurse practitioners and other researchers (Table 1). Mean years in practice was 13.3 years (+/− 9.9) with a range of 0 to 48 years. Due to concerns regarding level of experience and likely overlap with attendings in clinical telemedicine visits, trainees (fellows and residents) as well as other practitioners (nurses, social work, clinical research coordinators) were excluded from the clinical telemedicine results below.

**Table 1:** Respondent roles and years experience

**RESPONDENTS**	**N (%)**
Pediatric Rheumatologist	157 (70.4)
Other Subspecialists/Providers	3 (1.3)
Resident/Fellow Trainees	41 (18.4)
Advanced Practice Providers	6 (2.7)
Nurse	2 (0.08)
Social Worker	1 (0.04)
Other Research Staff	9 (4.0)
Did not answer	4 (1.8)
Total	223
**YEARS PRACTICE**	**Years**
Mean Years Practice (Total)	13.2 years
Range Years Practice	0 to 48 years
Number omitted/no answer	68

### Volumes of telemedicine use and types of Telehealth platforms

Prior to the COVID-19 pandemic, baseline use of telemedicine was minimal—85.9% of providers in this survey reported not conducting telemedicine visits. At the time of this survey, during the pandemic, only 2.0% of providers reported not having completed video visits over the last month. Volumes of video-visit use have also increased dramatically; 60.1% of providers have conducted greater than twenty direct provider-to patient video visits over the last month compared to 2.0% of providers prior to the pandemic. In terms of how providers are conducting telemedicine visits, similar numbers of providers utilize a free-standing telehealth platform only (37.4%) versus 36.1% that utilize a telehealth program that is either embedded or external to the electronic health record (EHR). 22.6% of providers reported using a telemedicine platform that was integrated with their EHR only.

### Telehealth use by modality

This survey item sought to evaluate changes (“Pre-Covid” to “current”) in the use of specific patient-care telehealth tools. Five forms of telehealth including patient portal usage, provider-to-provider telemedicine visits, provider-to-patient telemedicine visits, provider-to-patient telephone visits and electronic consults, revealed an increase in use from “Pre-Covid” to present times. The greatest increase seen in the use of provider to patient video visits which had eight-fold increase (*n* = 18 to *n* = 148) from “Pre-Covid” to “Current” use. Use of the patient portal “Pre-Covid” and “currently” was relatively stable with *n* = 119 using this telehealth technology pre-pandemic and *n* = 124 using currently. Similar numbers were seen amongst use of store and forward data with *n* = 102 “Pre-Covid” and *n* = 103 “Currently.” Other types of telehealth use described by survey respondents (as a free form response) included the use of telehealth for family meetings, patient education and teaching.

### Physical exam, patient and caregiver engagement, and assessment with use of telemedicine

Survey participants were queried regarding the ability to perform standard exam components reliably during a video visit. Though this survey was presented to rheumatology-focused providers, all exam components were included. The exam features reported as being most reliably completed by video visit (as determined by total number selected) were examination of the extremities, the musculoskeletal cervical exam, musculoskeletal hand, wrist and elbow, musculoskeletal knees and ankles, skin exam, and mental status. The exam components that ranked lowest were the cardiovascular, respiratory, gastrointestinal, strength, and neurologic examination (Table 2). Of note, 69.1% of survey participants reported utilizing a standardized exam approach during video visits and 86.5% of participants also reported engaging the patient or patient caregiver to help conduct components of the virtual exam. Of the standardized exam approaches, the vast majority of providers reported use of the Pediatric Gait Arms Legs and Spine (pGALS) [10] or a modified form, as well as other variations of standardized exams such as the Childhood Myositis Assessment Scale (CMAS) [11] or their own in-person clinical template adapted for telemedicine. In terms of how patient caregivers were included in the examination process, providers reported (in free-form response) that caregivers were asked to palpate, assess for warmth and passively range various joints, as well as other exam components such as identifying skin lesions, abdominal palpation, assessing for edema and lymph nodes, and directing the video camera for examination purposes. 36.7% of practitioners reported that patient engagement did not change via telemedicine and 38.0% of respondents did report that they felt that telemedicine moderately worsened patient engagement when compared to in-person clinical visits while 6.8% reported that telemedicine significantly worsened engagement. 18.4% of providers felt that telemedicine improved patient engagement. Additionally, a majority of providers (65.7%) felt that they were not able to elicit all the pertinent information needed to make a complete clinical assessment. Providers who indicated that they did not have all the pertinent information needed to make a clinical assessment were prompted to note what additional information was required. The free response data was not amenable to standard qualitative analysis, but providers commonly indicated the following as additional information needed: vitals, labs, limitations of exam including need for detailed exam (example: joint palpation, ausculatation) and concerns with reliability of exam (for example, patients have active arthritis not picked up via telemedicine exam).

**Table 2:** Exam components by percent selected “able to complete reliably”

Exam Component	Number selected as reliably done by video	(%)
MSK hands/wrists/elbows	101	62.7
MSK: cervical	100	62.1
Mental Status	95	59.0
MSK knees/ankles	91	56.5
Extremities	85	52.8
Skin	85	52.8
MSK: TMJ	75	46.6
Eye exam (external only)	71	44.1
MSK hips	60	37.3
Oral Exam	57	35.4
MSK Spine	53	32.9
MSK strength	47	29.2
Neurologic exam	21	13.0
Respiratory	14	8.7
GI exam	4	2.5
CV	3	1.9

### Telemedicine by encounter type

In considering appropriateness of telemedicine patient encounters, by type of encounter, the majority of providers selected routine follow up visits, encounters for injection teaching, and need for labs as appropriate visits for either in-person or telemedicine visits. The greatest number of providers selected the following encounter types as appropriate for in-person only: follow-up visit due to concern for flare, urgent follow up, or triage for anticipated hospitalization (Table 3).

**Table 3:** Encounter type by number selected as “in-person” appropriate only

Encounter Type	Number selected in-person only	% selected in-person only
Follow-up due to concern for flare	120	53.8%
New patient consultations	107	47.9%
Triage for anticipated hospitalization	106	47.5%
Patient concern for worsening condition	78	34.9%
Injection teaching	57	25.5%
Need for labs	22	9.8%
Routine follow-up visits	15	6.7%

### Impact of telemedicine on patient care

**Fig. 1:**
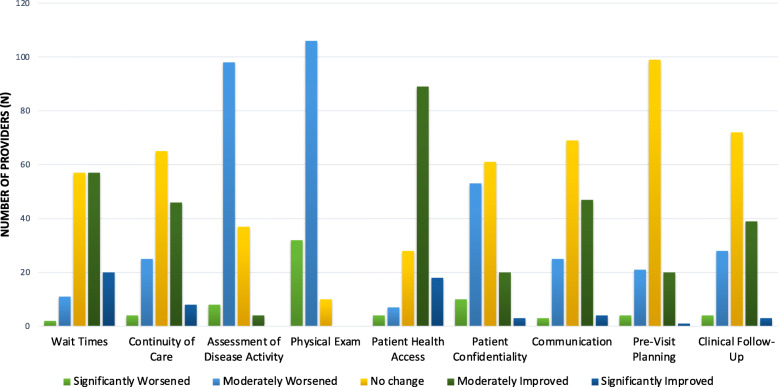
Assessment of clinical components of encounters by components

The following trends were observed regarding patient care and telemedicine. Patient wait times, continuity of care, patient health access, communication, pre-visit planning and clinical follow up were areas of care that were predominantly rated as no change to moderately or significantly improved secondary to telemedicine. In contrast, the majority of surveyed providers rated assessment of disease activity and physical exam as parts of clinical care that, in general, have been moderately worsened through use of telemedicine care delivery. In terms of adolescent and young adult confidentiality, there was no majority of opinion amongst providers; 41.5% of providers rated that confidentiality was not significantly changed whereas 36.1% of providers rated it as moderately worsened, and 13.6% reported that it was moderately improved (Fig. 1).

### Impact of telemedicine on provider burnout and satisfaction

Though most providers reported that telemedicine did not change their (self-perceived) level of burnout (54.0%), approximately a third (35.8%) of providers reported an increase in their level of burnout.

## Discussion

The use of telemedicine during the COVID-19 pandemic has been omnipresent and its potential effects on clinical care—for better or for worse—cannot be ignored. Pediatric rheumatologists have long struggled with ways to provide continuity and access to patients [12] and telemedicine may have a place in routine ambulatory care moving forward. Barriers to telemedicine use in pediatric ambulatory care have included limitations in policy and reimbursement, technology [13, 14] and understanding the appropriateness and potential impact on patient health outcomes. Due to sheer volume of ambulatory telemedicine use with COVID-19, we have the opportunity to understand the real-world use of telemedicine in multiple specialties including pediatric rheumatology. This brief study asked a large cohort of pediatric rheumatology providers to detail their real-world experience of telemedicine use during a time where telemedicine, for many providers, was the dominant modality of care. We were able to observe specific trends amongst clinical care domains including exam reliability, visit type appropriateness, and the ability to make a clinical assessment, but also determined potential areas of disagreement amongst providers surrounding adolescent confidentiality, patient engagement, and provider burnout. As providers, we have a responsibility to our patients to optimize the integration of telemedicine care and address equity and disparities, identify tele-amenable issues, and measure quality and safety [15].

With respect to visit type appropriateness, we found that in general, providers felt telemedicine was most appropriate for routine follow-up or if there were ancillary needs such as medication administration or lab result discussions. Generally, providers felt that urgent needs—whether flare of disease or patient concern regarding disease activity—were best suited for in-person clinical assessment. Interestingly, the majority of providers in this survey felt that components of the musculoskeletal exam, minus strength testing, were amongst the most reliable exam components done via video visit and the majority of providers were using a modified approach of standardized exams (such as the PGALS) with caregiver assistance to make their assessments. Whether other components (such as respiratory or skin exams) of the virtual exam are less reliable or that pediatric rheumatology providers feel most comfortable with the musculoskeletal exam by virtue of expertise in rheumatic disease is a question worth additional exploration. Regardless, due to the nature of pediatric rheumatic disease, virtualization of the standard musculoskeletal exam is necessary in pediatric rheumatology telemedicine visits [16]. Regardless of visit type appropriateness or ability to conduct a reliable exam, the majority of providers (65.7%) felt that they were not able to elicit all the needed information from a telemedicine visit to make a complete clinical assessment. Further investigation needs to be done to outline the components that contribute to these perceived shortfalls of the telemedicine clinical assessment. Areas of further investigation may include the impact of diagnosis and disease state, patient-reported outcomes in a virtual visit, and components of the exam that are not able to be reliably assessed via video. Ultimately, controlled clinical studies evaluating in-person versus telemedicine visits may be required to understand the complete impact of telemedicine-related factors on the ability to make a sound clinical assessment.

Regarding patient engagement, the results appeared split between no change and less patient engagement despite providers reporting that patient caregivers were directly participating in the physical assessment tasks. Over a third of providers felt that patient engagement was worse when compared to in person clinical visits. This is an important finding in a specialty which provides chronic disease management because improved patient engagement and shared decision-making have been suggested to correlate with improved health outcomes [17, 18]. Beyond health outcomes, there may be important correlates surrounding patient engagement and communication in the health care transition of patients from pediatric to adult rheumatic care [19].

An additional area of interest in pediatric rheumatology and in pediatrics in general, is teenage and young adult confidentiality. A benefit of telemedicine may include being able to take note of the patients’ environment and the potential implications of that environment on health, however, patients may have limited ability to be able to engage confidentially with their provider. During an in-person visit, there is no question of who is and is not in the exam room and it is less clear in a telemedicine visit. Though 41.5% of providers felt that confidentiality was not changed via telemedicine, over a third (36.1%) of providers felt that it was worsened when compared to an in-person visit. Providers must learn how to adapt their practice to ensure adolescent confidentiality and integrate new approaches to ensure this in telemedicine visits [20].

Lastly, this survey acknowledges the potential of telemedicine to impact physician burnout. Physician burnout is multifactorial, though there is some suggestion that COVID-19 has introduced new stressors [21, 22]. Given the rapid deployment of telemedicine, the change in the care delivery model, and the increase in volume of visits, telemedicine may inevitably be associated with provider workload or stress versus increases in provider productivity and worktime saved. Though burnout was increased in approximately a third of survey respondents, we cannot exclude other confounding factors and stressors related to the COVID-19 pandemic that occurred simultaneously with the increase in telemedicine care. The rapid implementation of technology in the health care system may place added stress on providers as they navigate novel roles of information technology and should not be overlooked. Further qualitative studies need to be performed to assess physician burnout in relation to telemedicine.

This survey was intended to identify specific clinical domains related to telehealth for further study and as such, is subject to several limitations. This study did not examine external factors such as lack of validated assessment tools, patient environment, provider telemedicine education, visit follow-up completion or intrinsic factors such as patient diagnosis, patient disease activity, and communication barriers. Further in-depth qualitative studies regarding the physician experience and additional clinical studies involving telemedicine visits in pediatric rheumatology are needed. Most of the providers surveyed in this study were pediatric rheumatologists which may have impacted specific findings related to reliability around certain aspects of the physical exam; therefore, these results may not directly apply to other pediatric subspecialties. In addition, this study does not involve the patient or caregiver perspective which is necessary when considering reliance on patient or caregiver reported outcomes via telemedicine. Lastly, this study does not separately evaluate the impact of COVID-19 or the rapidity of telemedicine deployment on the physician experience.

A potentially unique finding in this study is that most pediatric rheumatologists surveyed felt that telemedicine use increased patient health access. Understanding the impact of telemedicine on access and addressing disparities in care is vital; a major challenge in pediatric rheumatology remains patient access to care, and telemedicine may be one avenue to address this. However, social inequities may introduce unforeseen disparities of care in the application of telemedicine. This survey did not specifically address this, and further work, particularly on the patient-facing side, is needed to understand this implication of telemedicine care.

## Conclusion

This is the first professional organization-wide study that captures a large cohort of pediatric rheumatologists and their experience regarding telemedicine use in their day to day practice during the COVID-19 pandemic. We found that in general, providers felt that components of the musculoskeletal exam were able to be done reliably through telemedicine, yet interestingly the majority of providers felt that they were not able to generate a complete clinical assessment. We also found that perception of patient engagement and confidentiality varied when compared to in person clinical visits, and further work is needed to fully understand the potential impact on patient care. This study was completed during what has been a very stressful time for individuals and the hospital systems they operate within—follow-up work, particularly around the above findings, is key as telemedicine becomes incorporated into routine rheumatologic practice. These survey findings only begin to uncover the complexities of telemedicine care in rheumatology and further in-depth qualitative, patient-facing, and outcomes-focused work is needed in order to develop standardized telemedicine approaches to pediatric rheumatologic care.

## Supplementary Information

**Additional file 1.**

## Data Availability

The datasets during and/or analyzed during the current study available from the corresponding author on reasonable request.

## Abbreviations

CARRA: Childhood Arthritis and Rheumatology Research Alliance
