# Supplementary material for: Telemedicine use by pediatric rheumatologists during the COVID-19 pandemic
Source: Pediatr Rheumatol Online J. 2021 Jun 16;19:93. doi: 10.1186/s12969-021-00565-7 (PMC8206874; doi:10.1186/s12969-021-00565-7)
Supplement: Supplementary file 1 — Additional file 1. [file 12969_2021_565_MOESM1_ESM.pdf]

Introduction

The following survey will gather information regarding telehealth use in pediatric rheumatology during this unprecedented time. We appreciate your time, and if there are other specific comments you would like to make please utilize the free-form text box at the end of the survey.

Survey Research Information Sheet Link

* 1. Please indicate if you would like to participate in the survey.

- Yes, I would like to participate.
- No, I would like to opt out.

* 2. What is your primary role?

- Pediatric Rheumatologist
- Pediatric Rheumatology Fellow
- Pediatric Resident
- Nurse Practitioner
- Nurse
- Physician’s Assistant
- Other (please specify)

3. How many years have you practiced?

4. What is your overall comfort level with the use of technology?

0 (Poor) — 10 (Extremely Comfortable)

5. Which of the following best describes your use of telehealth technologies in routine patient care? (select all that apply):

	Pre-COVID	Currently
Patient portal with direct messaging	☐	☐
Provider-to-provider video visits (ex: Project Echo)	☐	☐
Patient/provider video visits	☐	☐
Patient-provider telephone visits	☐	☐
Electronic consults/referrals	☐	☐
Store and forward data review (ex: photos for dermatology review)	☐	☐
Other	☐	☐

Please specify Other if selected:

* 6. What type of telehealth program(s) do you use?

- ○ Integrated with the EHR (Electronic Health Record)
- ○ External (ex. Webex, Zoom, FaceTime, WhatsApp, etc.)
- ○ Both
- ○ None of the above

7. Which EHR patform do you use?

* 8. Which external platform(s) do you use for telehealth?

- [ ] Webex
- [ ] Zoom
- [ ] FaceTime
- [ ] WhatsApp
- [ ] Other (please specify)

9. Which EHR patform do you use?

10. Which external platform(s) do you use for telehealth?

- [ ] Webex
- [ ] Zoom
- [ ] FaceTime
- [ ] WhatsApp
- [ ] Other (please specify)

11. How many direct provider-to-patient video visits did you conduct pre-COVID?

- ( ) I did not conduct video visits.
- ( ) 1-5 per month
- ( ) 6-10 per month
- ( ) 11-15 per month
- ( ) 16-20 per month
- ( ) >20 visits per month

12. How many direct provider to patient video visits have you conducted over the last month?

- ◯ I have not conducted video visits.
- ◯ 1-5 visits
- ◯ 6-10 visits
- ◯ 11-15 visits
- ◯ 16-20 visits
- ◯ >20 visits

13. During a direct provider to patient video visit, what components of the exam are you able to reliably conduct as needed? (Please select all that apply)

- ☐ Oral exam
- ☐ Eye exam (external only)
- ☐ Skin exam (includes hair/nails)
- ☐ CV exam
- ☐ Respiratory
- ☐ GI exam
- ☐ Extremities: cyanosis/edema
- ☐ MSK TMJ exam
- ☐ MSK cervical exam
- ☐ MSK hands/wrists/elbows
- ☐ MSK knees/ankles
- ☐ MSK spine
- ☐ MSK hips
- ☐ MSK strength
- ☐ Neurologic exam
- ☐ Mental Status
- ☐ Other (please describe)

14. Do you utilize a standardized physical exam approach for video visits (ex: PGALS)?

- ◯ No
- ◯ Yes (please describe)

15. Have you ever engaged the patient caregiver or patient to conduct the exam components for these virtual visits (this may include directed exam components such as palpation, ranging joints, identifying specific skin lesions, etc):

- ◯ No
- ◯ Yes (please describe)

16. Do you think telehealth visits compared to in person visits has changed the way you engage with your patients < 18 years of age?

- Telehealth visits significantly worsen patient engagement compared to in person visits
- Telehealth visits worsen patient engagement compared to in person visits
- Telehealth visits are the same regarding patient engagement compared to in person visits
- Telehealth visits improve patient engagement compared to in person visits
- Telehealth visits significantly improve patient engagement compared to in person visits

17. Does your medical center use telemedicine visits to conduct research?

- Yes
- No
- Unsure

18. In considering the majority of your video visits, have you able to elicit all the pertinent information needed to make a complete clinical assessment?

- Yes
- No (Please specify what additional information was required— ex. labs, vitals, exam components, nursing assistance, etc.)

19. What type of patient encounters do you think are appropriately done by visit type?

	In-Person	Telemedicine	Either/Both
Injection Teaching	○	○	○
Routine follow-up visits	○	○	○
Urgent follow-up Visits	○	○	○
New patient consultations	○	○	○
Follow-up due to concern for flare	○	○	○
Triage for anticipated hospitalization	○	○	○
Need for labs	○	○	○
Patient concern for worsening condition	○	○	○

20. Has the utilization of health technologies, such as video visits, changed your self-identified level of burnout?

- ◯ Increased burnout
- ◯ Decreased burnout
- ◯ No change in burnout

21. How has telemedicine affected the following (select one per item):

	Significantly Worsened	Moderately Worsened	No Change	Moderately Improved	Significantly Improved
Wait Times	◯	◯	◯	◯	◯
Continuity of care	◯	◯	◯	◯	◯
Assessment of disease activity	◯	◯	◯	◯	◯
Physical exam	◯	◯	◯	◯	◯
Patient Health Access	◯	◯	◯	◯	◯
Patient Confidentiality with Teenagers/Young Adults	◯	◯	◯	◯	◯
Communication	◯	◯	◯	◯	◯
Pre-Visit Planning	◯	◯	◯	◯	◯
Clinical Follow-Up	◯	◯	◯	◯	◯

22. What is your overall level of satisfaction of the recent use of video visits to provide direct patient care?

0 - Not Satisfied 100 - Extremely Satisfied

23. Please provide any additional comments regarding your video visit experience:

Thank you for your time. If there are specific items that you would like to address specifically with the survey leaders, please email Rajdeep Pooni at rpooni@stanford.edu.
